# Polymorphism of bis­(1,3-benzo­thia­zol-2-yl) tri­thio­carbonate

**DOI:** 10.1107/S2056989020008105

**Published:** 2020-06-23

**Authors:** Kevin Kafuta, Christopher Golz, Manuel Alcarazo

**Affiliations:** a Georg-August-Universität Göttingen, Institut für Organische und Biomolekulare Chemie, Tammannstrasse 2, D-37077 Göttingen, Germany

**Keywords:** crystal structure, tri­thio­carbonates, benzo­thia­zole, polymorphism, Hirshfeld surface analysis

## Abstract

The polymorphism of the title compound, C_15_H_8_N_2_S_5_, is reported, in which the (*syn*,*syn*) and (*syn*,*anti*) conformers simultaneously crystallized from a chloro­form solution. The complete mol­ecule of the (*syn*,*syn*) form is generated by a crystallographic twofold axis. The geometries of both conformers are compared in detail, revealing no significant differences in bond lengths, despite different bond angles because of the conformational changes. Analysis of the inter­molecular inter­actions, aided by Hirshfeld surfaces, shows distinctive S⋯S and S⋯N contacts only for the (*syn*,*anti*) conformer. Aromatic π–π-stacking inter­actions are found for both conformers, which occur for the (*syn*,*anti*) conformer between pairs of mol­ecules, but are continuous stacks in the (*syn*,*syn*) conformer. Non merohedral twinning was found for the crystal of the (*syn*,*anti*) conformer used for data collection.

## Abstract 2

Bis(benzo­thia­zol-2-yl)tri­thio­carbonate, C_15_H_8_N_2_S_5_, crystallizes in two visually distinguishable polymorphs, each containing a different conformer of the title compound and featuring different kinds of intra- and inter­molecular S⋯S, S⋯N and π–π inter­actions.

## Chemical context   

Acyclic tri­thio­carbonates are important functional groups in several areas ranging from materials science and synthetic chemistry to pharmaceutics (Kazemi *et al.*, 2018 ▸). Notably, their use as reagents in reversible addition–fragmentation chain-transfer (RAFT) free radical polymerization appears relevant since the relative stability of the conformers might have an influence on the stereochemistry of the obtained polymer (Huang *et al.*, 2018 ▸). Earlier studies on the conformational properties of perfluoro­dimethyl tri­thio­carbonate based on gas electron diffraction and Raman spectroscopy (Hermann *et al.*, 2000 ▸) show clear dependency of the solvent and aggregate state: The (*syn*,*syn*) conformer is predominant (84%) in the gas phase, as a liquid the distribution is almost equal [60% (*syn*,*syn*)], while in solution and with increasing polarity of the solvent, the ratio of the (*syn*,*anti*) conformer increases. The herein reported conformational polymorphism allows further structural comparison between tri­thio­carbonate conformers by X-ray diffraction analysis.
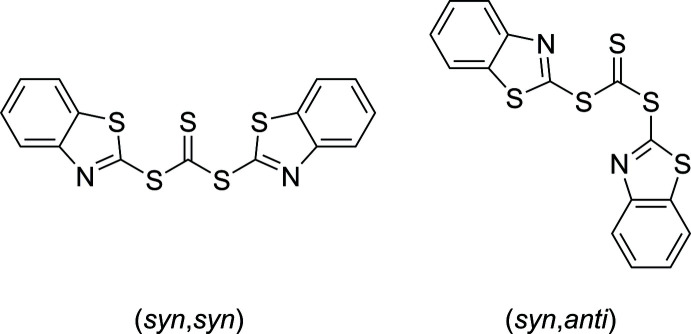


## Structural commentary   

**Figure 1:**
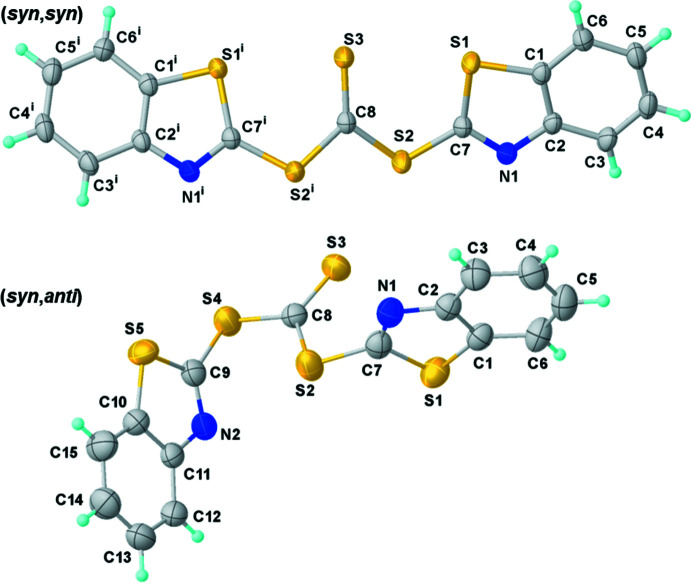
Mol­ecular structures and labelling schemes for the (*syn*,*syn*) (top) and (*syn*,*anti*) (bottom) polymorphs with displacement ellipsoids at the 50% probability level. Symmetry code: (i) −*x*, +*y*, 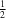 − *z*.

The (*syn*,*syn*) conformer crystallizes from chloro­form solution in space group *Pbcn*. The asymmetric unit contains half of the mol­ecule, with a crystallographic twofold axis passing through S3—C8 generating the complete mol­ecule. The mol­ecule is slightly twisted in a propeller-like shape, the twist introduced by the C7—S2—C8—S3 torsion angle of 24.46 (12)°, thus deviating from the idealized *syn* geometry of 0° (Fig. 1 ▸).

The (*syn*,*anti*) conformer also crystallizes from chloro­form solution, but in space group *P* 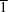. The *syn* and *anti* conformations of each half of the mol­ecule are closer to the idealized geometry with torsion angles C7—S2—C8—S3 of 2.17 (13) and C9—S4—C8—S3 of −174.93 (9)°.

Inter­estingly, the thio­carbonyl and thia­zol moieties in the (*syn*,*anti*) conformer are oriented almost perpendicular to each other, with S1—C7—S2—C8 and S5—C9—S4—C8 torsion angles close to 90° [–87.25 (10) and 104.26 (10)°, respectively]. In the (*syn*,*syn*) conformer, the thio­carbonyl and thia­zol groups are almost in plane, apart from the propeller-like twist: the respective torsion angle S1—C7—S2—C8 is −2.4 (2)°, resulting in relative close proximity for the thio­carbonyl S1 and thia­zol S3 atoms. This S1⋯S3 distance of 3.106 (1) Å, which is considerably shorter than the sum of van der Waals radii (3.78 Å; Alvarez, 2013 ▸), indicates possible attractive chalcogenic inter­actions. Comparable T-shaped geometries around sulfur were observed by our group for dihalosulfuranes (Talavera *et al.*, 2015 ▸; Peña *et al.*, 2017 ▸; Averesch *et al.*, 2019 ▸), where the inter­action is even more pronounced because of the electronically depleted sulfur atoms.

The bond lengths in both conformers show no significant differences that would correspond to the changed bond angles in respect of hyperconjugative effects.

## Supra­molecular features   

**Figure 2:**
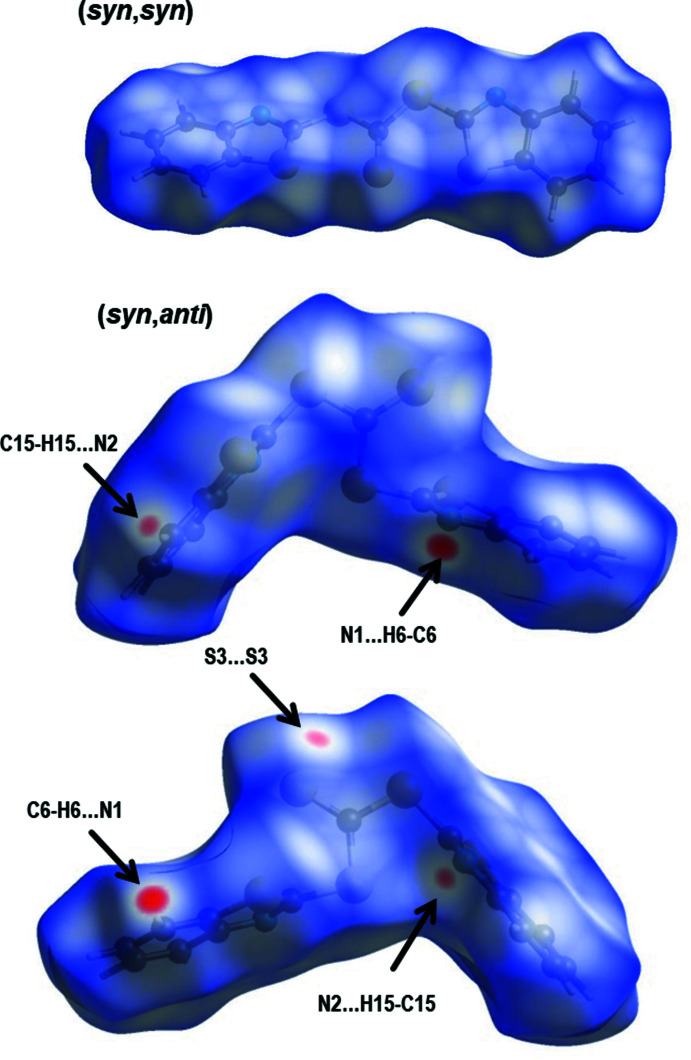
Hirshfeld surfaces mapped over *d*_norm_ for the (*syn*,*syn*) (top) and (*syn*,*anti*) (bottom) conformers in opposite views. Mapped ranges of 0.0097 to 1.0175 and −0.1520 to 1.2170 for (*syn*,*syn*) and (*syn*,*anti*), respectively.

**Figure 3:**
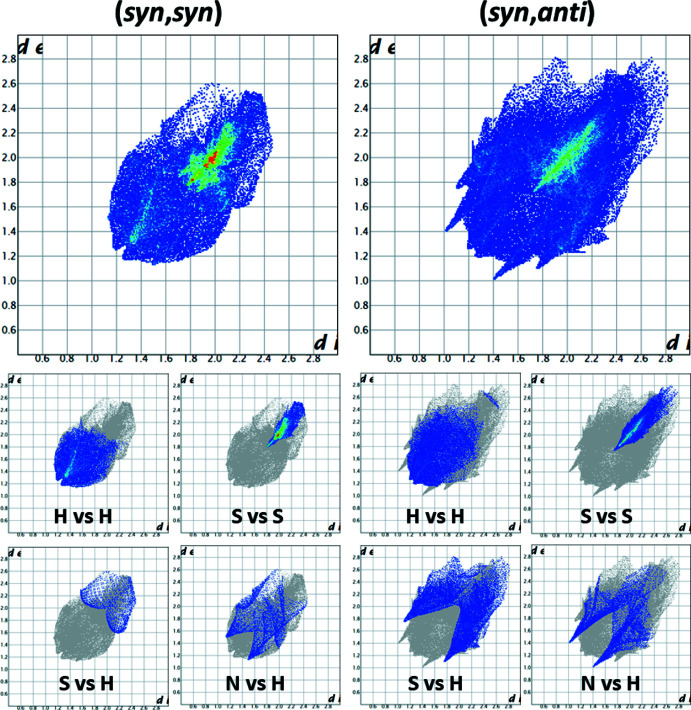
Full fingerprint plot (top) and decomposed plots (bottom) showing exclusive element element contacts.

To investigate the supra­molecular features, the Hirshfeld surface (Spackman & Jayatilaka, 2009 ▸) was calculated for both conformers using *CrystalExplorer17* (Turner *et al.*, 2017 ▸). The resulting Hirshfeld surfaces mapped over *d*_norm_ heatmaps for the (*syn*,*syn*) and (*syn*,*anti*) conformers are depicted in Fig. 2 ▸ and the corresponding fingerprint plots are shown in Fig. 3 ▸. Observation of the heatmap and the features of the fingerprint plots yields one apparent conclusion: the (*syn*,*syn*) conformer has no distinctive contacts while the surface for the (*syn*,*anti*) conformer features in total five hot spots, which reappear as sharp features in the fingerprint plot (Fig. 3 ▸). Those contacts are identified as two C—H⋯N hydrogen bonds (Table 1 ▸) between N1 and C6 and N2 and C15 with lengths of 3.467 (2) and 3.552 (2) Å, respectively. This is in the range of other C—H⋯N hydrogen bonds reported previously (Mambanda *et al.* 2007 ▸; Pingali *et al.*, 2014 ▸). The fifth contact is a symmetric S⋯S inter­action between S3 and its adjacent symmetry-equivalent clone, with a distance of 3.509 (1) Å. The relative contributions of various contacts to the Hirshfeld surface are given in Table 2 ▸.

**Figure 4:**
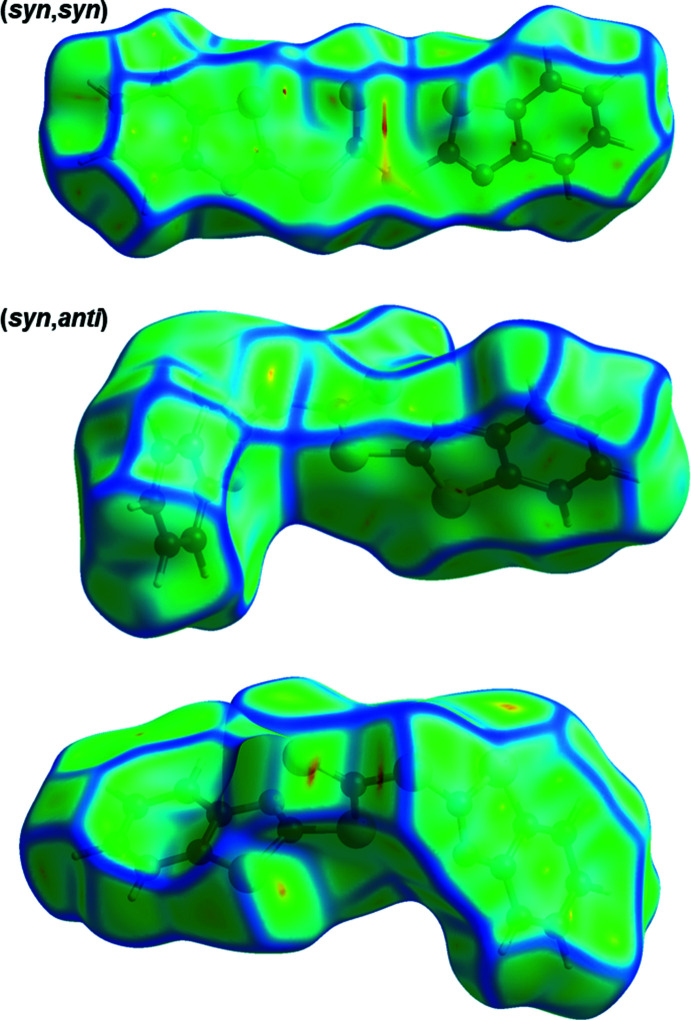
Hirshfeld surface mapped over curvedness for the (*syn*,*syn*) (top) and (*syn*,*anti*) (bottom) conformers in opposite views.

**Figure 5:**
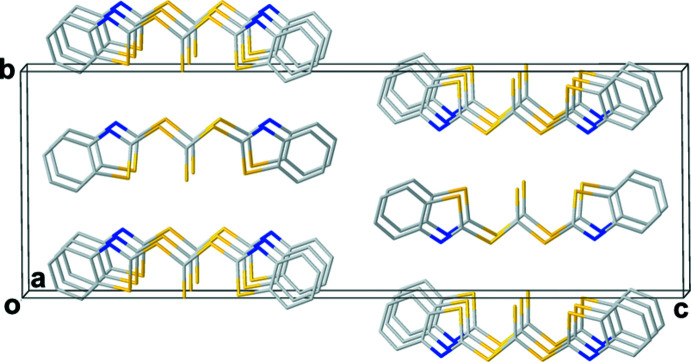
Packing diagram for (*syn*,*syn*) displaying the herringbone motif.

**Figure 6:**
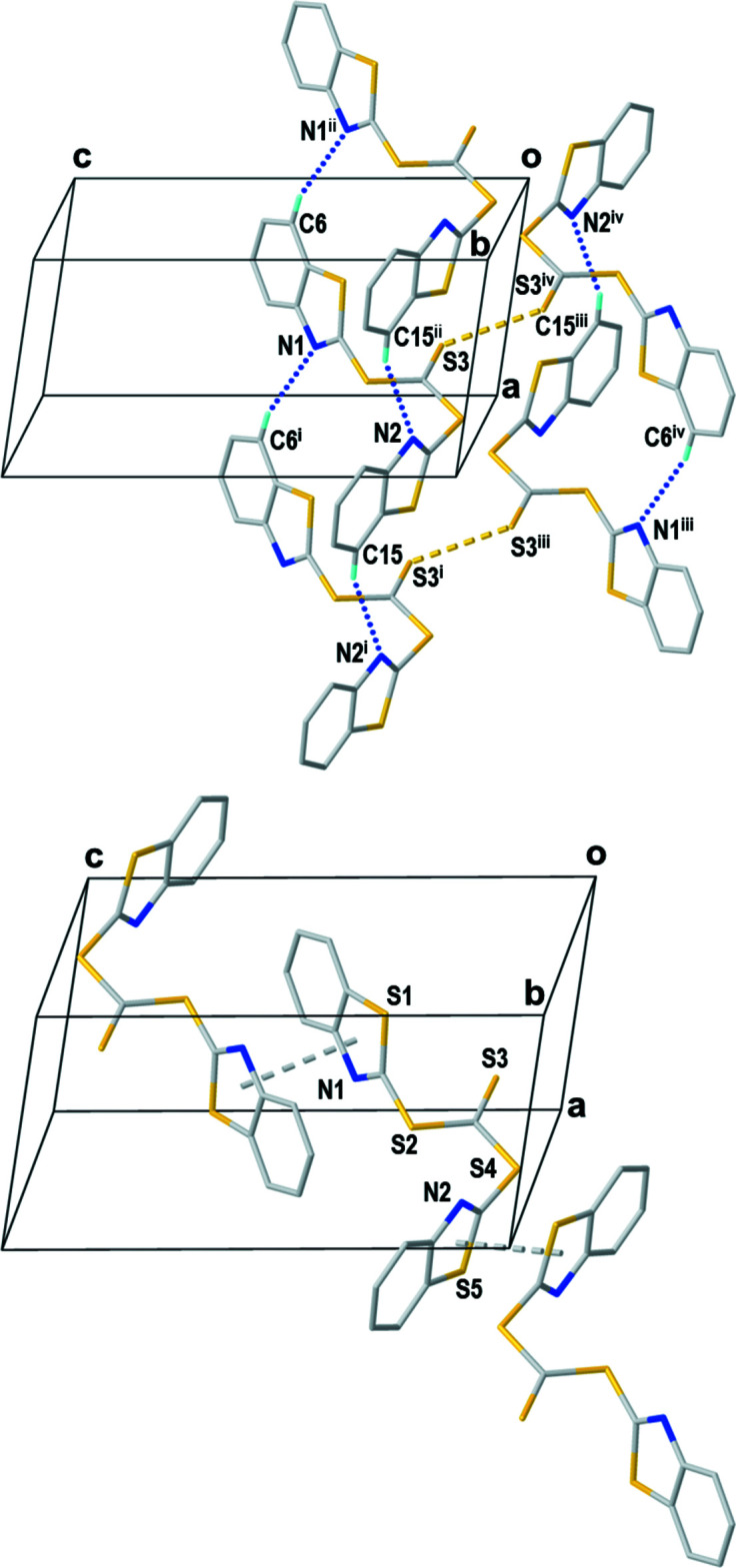
Packing diagram displaying all S⋯S contacts (yellow stippled bonds) and C—H⋯N hydrogen bonds (blue balled bonds) and separate packing diagram displaying π–π inter­actions (grey stippled bonds). Only hydrogen atoms participating in hydrogen bonds are shown. Symmetry codes: (i) 1 + *x*, +*y*, +*z*; (ii) −1 + *x*, +*y*, +*z*; (iii) 2 − *x*, 1 − *y*, −*z*; (iv) 1 − *x*, 1 − *y*, −*z*.

The Hirshfeld surface mapped over curvedness (Fig. 4 ▸) indicates π–π inter­actions by wide flat areas on one side of each benzo­thia­zol unit. Packing diagrams of the (*syn*,*syn*) (Fig. 5 ▸) and (*syn*,*anti*) (Fig. 6 ▸) conformers show the parallel arrangement of adjacent benzo­thia­zol groups, which come in pairs (*syn*,*anti*) or in a continuous herringbone motif (*syn*,*syn*). The separation between the benzo­thia­zol planes (defined by C1–C7/N1/S1 or C9–C15/N2/S5) are similar with distances of 3.54 Å in the (*syn*,*syn*) conformer and 3.43 and 3.58 Å in the (*syn*,*anti*) conformer.

## Database survey   

A search in the CSD (version 5.41, update of November 2019; Groom *et al.*, 2016 ▸) for non-cyclic and non-oxidized tri­thio­carbonates produced 20 results, of which only one (refcode XUBNAJ; Sotofte & Senning, 2001 ▸) adopts a (*syn*,*anti*) conformation. There is one outlier neither close to a (*syn*,*syn*) nor a (*syn*,*anti*) conformation, which is chemically a thio­anhydride (XISSAU; Weber *et al.*, 2008 ▸). All other results are tri­thio­carbonates with a (*syn*,*syn*) conformation, which appears to be the predominant form. A substructure search for benzo­thia­zoles and thia­zoles yielded large numbers of hits (1500 and 2200, respectively). A comparable polymorphism with thia­zolo­thia­zols (Schneider *et al.*, 2015 ▸) was reported with inter­plane separations around 3.45 Å, as well as arrangements in pairs of π–π inter­actions for one polymorph and a herringbone motif in the other, closely matching our observations.

## Synthesis and crystallization   

The title compound was initially isolated in small amounts as a side product and crystallized from chloro­form solution in an NMR tube, where two crystalline species could be identified visually: (*syn*,*syn*) in the form of orange needles and (*syn*,*anti*) as orange blocks.

The synthesis of the title compound is based on a literature procedure (Runge *et al.*, 1962 ▸). Benzo­thia­zole-2-thiol (500 mg, 2.99 mmol, 1.0 eq.) and sodium hydroxide (179 mg, 4.48 mmol, 1.5 eq.) were dissolved in water (30 ml). Thio­phosgene (165 mg, 1.44 mmol, 0.48 eq.) was added dropwise at room temperature. After complete addition, the solution was stirred for 15 minutes. Brine solution was added, the reaction mixture extracted with ethyl acetate and the combined organic phases dried over sodium sulfate. The solvent was removed *in vacuo* to yield the crude product as an orange solid (518 mg). After recrystallization from boiling benzene solution the pure product was obtained as orange crystals (432 mg, 1.15 mmol, 77%).

The melting range is 420–423 K, as measured on a Büchi M-560.

NMR spectra recorded on a Bruker Avance III HD 300 and chemical shifts are given in parts per million. ^1^H NMR (300 MHz, CDCl_3_): 8.20–8.17 (*m*, 1H), 7.99–7.96 (*m*, 1H), 7.62–7.51 (*m*, 2H). ^13^C-NMR (300 MHz, CDCl_3_): 212.5 (C), 156.5 (C), 152.8 (C), 138.0 (C), 126.94 (CH), 126.85 (CH), 124.4 (CH), 121.7 (CH).

High-resolution mass spectrometry was carried out on a Bruker maXis ESI–QTOF–MS. Calculated for C_15_H_8_S_5_N_2_+H^+^: 376.9364, found: 376.9364; calculated for C_15_H_8_S_5_N_2_+Na^+^: 398.9183, found: 398.9185.

## Refinement   

Crystal data, data collection and structure refinement details are summarized in Table 3 ▸. All aromatic hydrogen atoms were placed geometrically (C—H = 0.93 Å) and refined using a riding model with *U*_iso_(H) = 1.2*U*_iso_(C).

Non merohedral twinning was found for the crystal of the (*syn*,*anti*) conformer used for data collection. The twin domain transformation matrix was found to be (0.996, −0.141, −0.031/0.331, 0977, −0.106/0.235, 0.130, 0.958). Data integration was carried out using both domains with a final twin batch scale factor of 0.1211 (17).

## Supplementary Material

Crystal structure: contains datablock(s) global, sa, ss. DOI: 10.1107/S2056989020008105/hb7924sup1.cif

Structure factors: contains datablock(s) ss. DOI: 10.1107/S2056989020008105/hb7924sssup3.hkl

Structure factors: contains datablock(s) sa. DOI: 10.1107/S2056989020008105/hb7924sasup4.hkl

Supporting information file. DOI: 10.1107/S2056989020008105/hb7924sssup4.cml

CCDC references: 2010358, 2010357

Additional supporting information:  crystallographic information; 3D view; checkCIF report

## Figures and Tables

**Table 1:** Hydrogen-bond geometry (Å, °) for the (*syn*,*anti*) conformer[Chem scheme1]

*D*—H⋯*A*	*D*—H	H⋯*A*	*D*⋯*A*	*D*—H⋯*A*
C6—H6⋯N1^i^	0.93	2.58	3.467 (2)	161
C15—H15⋯N2^ii^	0.93	2.68	3.552 (2)	157

Symmetry codes: (i) 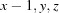; (ii) 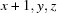.

**Table 2:** Relative element–element contributions to the Hirshfeld surface (in %). Asymmetric contacts include reciprocal contributions

Contact	(*syn*,*syn*)	(*syn*,*anti*)
S⋯S	22.9	16.1
S⋯N	4.6	0.9
S⋯C	6.2	11.5
S⋯H	2.7	17.4
N⋯N	0.4	0.2
N⋯C	2.6	3.3
N⋯H	8.4	9.7
C⋯C	11.6	3.0
C⋯H	9.6	16.5
H⋯H	31.0	21.4

**Table 3:** Experimental details

	(*syn*,*syn*)	(*syn*,*anti*)
Crystal data		
Chemical formula	C_15_H_8_N_2_S_5_	C_15_H_8_N_2_S_5_
*M*_r_	376.53	376.53
Crystal system, space group	Orthorhombic, *P* *b* *c* *n*	Triclinic, *P* 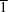
Temperature (K)	299	299
*a*, *b*, *c* (Å)	3.9458 (15), 11.434 (3), 33.366 (11)	6.4976 (5), 9.5593 (12), 13.1962 (11)
α, β, γ (°)	90, 90, 90	80.576 (5), 82.312 (3), 86.288 (5)
*V* (Å^3^)	1505.4 (9)	800.55 (14)
*Z*	4	2
Radiation type	Mo *K*α	Mo *K*α
μ (mm^−1^)	0.76	0.72
Crystal size (mm)	0.24 × 0.07 × 0.02	0.44 × 0.35 × 0.28

Data collection		
Diffractometer	Bruker D8 Venture Dual Source	Bruker D8 Venture Dual Source
Absorption correction	Multi-scan (*SADABS*; Bruker, 2016 ▸)	Multi-scan (*TWINABS*; Bruker, 2012 ▸)
*T*_min_, *T*_max_	0.248, 0.336	0.256, 0.372
No. of measured, independent and observed [*I* > 2σ(*I*)] reflections	17286, 2147, 1708	4846, 4846, 4296
*R*_int_	0.048	–
(sin θ/λ)_max_ (Å^−1^)	0.699	0.714

Refinement		
*R*[*F* ^2^ > 2σ(*F* ^2^)], *wR*(*F* ^2^), *S*	0.051, 0.110, 1.10	0.036, 0.099, 1.03
No. of reflections	2147	4846
No. of parameters	101	200
H-atom treatment	H-atom parameters constrained	H-atom parameters constrained
Δρ_max_, Δρ_min_ (e Å^−3^)	0.32, −0.34	0.45, −0.43

Computer programs: *APEX3* and *SAINT* (Bruker, 2016 ▸), *SHELXT* (Sheldrick, 2015*a* ▸), *SHELXL* (Sheldrick, 2015*b* ▸) and *OLEX2* (Dolomanov *et al.*, 2009 ▸).

## supplementary crystallographic information

### Bis(1,3-benzothiazol-2-yl) trithiocarbonate (ss) Crystal data

**Table:**

C_15_H_8_N_2_S_5_	*D*_x_ = 1.661 Mg m^−^^3^
*M**_r_* = 376.53	Melting point: 420 K
Orthorhombic, *P**b**c**n*	Mo *K*α radiation, λ = 0.71073 Å
*a* = 3.9458 (15) Å	Cell parameters from 744 reflections
*b* = 11.434 (3) Å	θ = 2.4–23.7°
*c* = 33.366 (11) Å	µ = 0.76 mm^−^^1^
*V* = 1505.4 (9) Å^3^	*T* = 299 K
*Z* = 4	Needle, orange
*F*(000) = 768	0.24 × 0.07 × 0.02 mm

### Bis(1,3-benzothiazol-2-yl) trithiocarbonate (ss) Data collection

**Table:**

Bruker D8 Venture Dual Source diffractometer	1708 reflections with *I* > 2σ(*I*)
Detector resolution: 8.33 pixels mm^-1^	*R*_int_ = 0.048
φ and ω scans	θ_max_ = 29.8°, θ_min_ = 2.4°
Absorption correction: multi-scan (SADABS; Bruker, 2016)	*h* = −5→5
*T*_min_ = 0.248, *T*_max_ = 0.336	*k* = −15→15
17286 measured reflections	*l* = −46→46
2147 independent reflections	

### Bis(1,3-benzothiazol-2-yl) trithiocarbonate (ss) Refinement

**Table:**

Refinement on *F*^2^	Primary atom site location: dual
Least-squares matrix: full	Hydrogen site location: inferred from neighbouring sites
*R*[*F*^2^ > 2σ(*F*^2^)] = 0.051	H-atom parameters constrained
*wR*(*F*^2^) = 0.110	*w* = 1/[σ^2^(*F*_o_^2^) + (0.0292*P*)^2^ + 2.5718*P*] where *P* = (*F*_o_^2^ + 2*F*_c_^2^)/3
*S* = 1.10	(Δ/σ)_max_ = 0.001
2147 reflections	Δρ_max_ = 0.32 e Å^−^^3^
101 parameters	Δρ_min_ = −0.34 e Å^−^^3^
0 restraints	

### Bis(1,3-benzothiazol-2-yl) trithiocarbonate (ss) Special details

**Table:**

Geometry. All esds (except the esd in the dihedral angle between two l.s. planes) are estimated using the full covariance matrix. The cell esds are taken into account individually in the estimation of esds in distances, angles and torsion angles; correlations between esds in cell parameters are only used when they are defined by crystal symmetry. An approximate (isotropic) treatment of cell esds is used for estimating esds involving l.s. planes.

### Bis(1,3-benzothiazol-2-yl) trithiocarbonate (ss) Fractional atomic coordinates and isotropic or equivalent isotropic displacement parameters (Å^2^)

**Table:**

	*x*	*y*	*z*	*U*_iso_*/*U*_eq_	
S1	0.3107 (2)	0.53882 (6)	0.33530 (2)	0.03516 (19)	
S2	−0.0018 (2)	0.75692 (6)	0.29112 (2)	0.0406 (2)	
S3	0.000000	0.52046 (9)	0.250000	0.0464 (3)	
N1	0.2014 (7)	0.7473 (2)	0.36435 (6)	0.0339 (5)	
C1	0.4357 (7)	0.5675 (2)	0.38429 (7)	0.0303 (5)	
C2	0.3531 (7)	0.6823 (2)	0.39456 (7)	0.0303 (5)	
C3	0.4219 (8)	0.7232 (3)	0.43318 (7)	0.0380 (7)	
H3	0.364207	0.798967	0.440712	0.046*	
C4	0.5772 (8)	0.6486 (3)	0.45974 (8)	0.0417 (7)	
H4	0.626489	0.674805	0.485467	0.050*	
C5	0.6622 (8)	0.5349 (3)	0.44906 (8)	0.0419 (7)	
H5	0.766095	0.486440	0.467776	0.050*	
C6	0.5954 (8)	0.4927 (3)	0.41122 (8)	0.0365 (6)	
H6	0.654756	0.416888	0.403921	0.044*	
C7	0.1712 (7)	0.6830 (2)	0.33240 (7)	0.0308 (5)	
C8	0.000000	0.6615 (3)	0.250000	0.0302 (8)	

### Bis(1,3-benzothiazol-2-yl) trithiocarbonate (ss) Atomic displacement parameters (Å^2^)

**Table:**

	*U*^11^	*U*^22^	*U*^33^	*U*^12^	*U*^13^	*U*^23^
S1	0.0508 (4)	0.0307 (3)	0.0240 (3)	0.0060 (3)	−0.0060 (3)	−0.0048 (2)
S2	0.0647 (5)	0.0311 (3)	0.0261 (3)	0.0092 (4)	−0.0079 (3)	−0.0031 (2)
S3	0.0809 (9)	0.0299 (5)	0.0284 (4)	0.000	−0.0129 (5)	0.000
N1	0.0472 (14)	0.0301 (11)	0.0244 (9)	0.0004 (11)	−0.0001 (10)	−0.0024 (8)
C1	0.0335 (14)	0.0346 (13)	0.0228 (10)	−0.0035 (11)	0.0007 (10)	−0.0013 (9)
C2	0.0366 (15)	0.0326 (13)	0.0216 (10)	−0.0048 (12)	0.0010 (10)	−0.0016 (9)
C3	0.0514 (18)	0.0395 (15)	0.0231 (11)	−0.0053 (13)	0.0025 (11)	−0.0053 (10)
C4	0.0486 (18)	0.0530 (18)	0.0235 (11)	−0.0101 (15)	−0.0047 (12)	−0.0022 (11)
C5	0.0489 (18)	0.0495 (17)	0.0275 (12)	−0.0040 (15)	−0.0064 (12)	0.0057 (12)
C6	0.0428 (16)	0.0357 (14)	0.0310 (12)	0.0030 (13)	−0.0057 (11)	0.0010 (10)
C7	0.0388 (15)	0.0318 (12)	0.0218 (10)	−0.0001 (12)	−0.0002 (10)	−0.0009 (9)
C8	0.035 (2)	0.0332 (18)	0.0227 (14)	0.000	−0.0008 (14)	0.000

### Bis(1,3-benzothiazol-2-yl) trithiocarbonate (ss) Geometric parameters (Å, º)

**Table:**

S1—C1	1.739 (2)	C2—C3	1.397 (3)
S1—C7	1.741 (3)	C3—H3	0.9300
S2—C7	1.755 (3)	C3—C4	1.375 (4)
S2—C8	1.753 (2)	C4—H4	0.9300
S3—C8	1.612 (4)	C4—C5	1.389 (4)
N1—C2	1.388 (3)	C5—H5	0.9300
N1—C7	1.300 (3)	C5—C6	1.377 (4)
C1—C2	1.395 (4)	C6—H6	0.9300
C1—C6	1.392 (4)		
			
C1—S1—C7	87.88 (12)	C5—C4—H4	119.2
C8—S2—C7	108.23 (13)	C4—C5—H5	119.4
C7—N1—C2	109.4 (2)	C6—C5—C4	121.1 (3)
C2—C1—S1	110.02 (19)	C6—C5—H5	119.4
C6—C1—S1	128.3 (2)	C1—C6—H6	121.2
C6—C1—C2	121.7 (2)	C5—C6—C1	117.6 (3)
N1—C2—C1	115.2 (2)	C5—C6—H6	121.2
N1—C2—C3	125.1 (2)	S1—C7—S2	128.53 (14)
C1—C2—C3	119.7 (2)	N1—C7—S1	117.46 (19)
C2—C3—H3	120.9	N1—C7—S2	114.0 (2)
C4—C3—C2	118.3 (3)	S2—C8—S2^i^	103.00 (19)
C4—C3—H3	120.9	S3—C8—S2	128.50 (10)
C3—C4—H4	119.2	S3—C8—S2^i^	128.50 (10)
C3—C4—C5	121.6 (2)		
			
S1—C1—C2—N1	−1.3 (3)	C4—C5—C6—C1	−0.8 (5)
S1—C1—C2—C3	178.1 (2)	C6—C1—C2—N1	178.7 (3)
S1—C1—C6—C5	−178.4 (2)	C6—C1—C2—C3	−1.9 (4)
N1—C2—C3—C4	−179.3 (3)	C7—S1—C1—C2	1.6 (2)
C1—S1—C7—S2	176.9 (2)	C7—S1—C1—C6	−178.3 (3)
C1—S1—C7—N1	−1.8 (2)	C7—S2—C8—S2^i^	−155.54 (12)
C1—C2—C3—C4	1.4 (4)	C7—S2—C8—S3	24.46 (12)
C2—N1—C7—S1	1.3 (3)	C7—N1—C2—C1	0.0 (4)
C2—N1—C7—S2	−177.49 (19)	C7—N1—C2—C3	−179.3 (3)
C2—C1—C6—C5	1.6 (4)	C8—S2—C7—S1	−2.4 (2)
C2—C3—C4—C5	−0.6 (5)	C8—S2—C7—N1	176.3 (2)
C3—C4—C5—C6	0.3 (5)		

Symmetry code: (i) −*x*, *y*, −*z*+1/2.

### (sa) Crystal data

**Table:**

C_15_H_8_N_2_S_5_	*Z* = 2
*M**_r_* = 376.53	*F*(000) = 384
Triclinic, *P*1	*D*_x_ = 1.562 Mg m^−^^3^
*a* = 6.4976 (5) Å	Mo *K*α radiation, λ = 0.71073 Å
*b* = 9.5593 (12) Å	Cell parameters from 1193 reflections
*c* = 13.1962 (11) Å	θ = 3.2–27.5°
α = 80.576 (5)°	µ = 0.72 mm^−^^1^
β = 82.312 (3)°	*T* = 299 K
γ = 86.288 (5)°	Block, yellow
*V* = 800.55 (14) Å^3^	0.44 × 0.35 × 0.28 mm

### (sa) Data collection

**Table:**

Bruker D8 Venture Dual Source diffractometer	4846 independent reflections
Detector resolution: 8.33 pixels mm^-1^	4296 reflections with *I* > 2σ(*I*)
φ and ω scans	θ_max_ = 30.5°, θ_min_ = 2.9°
Absorption correction: multi-scan (*TWINABS*; Bruker, 2012)	*h* = −9→9
*T*_min_ = 0.256, *T*_max_ = 0.372	*k* = −13→13
4846 measured reflections	*l* = 0→18

### (sa) Refinement

**Table:**

Refinement on *F*^2^	Primary atom site location: dual
Least-squares matrix: full	Hydrogen site location: inferred from neighbouring sites
*R*[*F*^2^ > 2σ(*F*^2^)] = 0.036	H-atom parameters constrained
*wR*(*F*^2^) = 0.099	*w* = 1/[σ^2^(*F*_o_^2^) + (0.0452*P*)^2^ + 0.2258*P*] where *P* = (*F*_o_^2^ + 2*F*_c_^2^)/3
*S* = 1.03	(Δ/σ)_max_ < 0.001
4846 reflections	Δρ_max_ = 0.45 e Å^−^^3^
200 parameters	Δρ_min_ = −0.43 e Å^−^^3^
0 restraints	

### (sa) Special details

**Table:**

Geometry. All esds (except the esd in the dihedral angle between two l.s. planes) are estimated using the full covariance matrix. The cell esds are taken into account individually in the estimation of esds in distances, angles and torsion angles; correlations between esds in cell parameters are only used when they are defined by crystal symmetry. An approximate (isotropic) treatment of cell esds is used for estimating esds involving l.s. planes.
Refinement. Refined as a 2-component twin.

### (sa) Fractional atomic coordinates and isotropic or equivalent isotropic displacement parameters (Å^2^)

**Table:**

	*x*	*y*	*z*	*U*_iso_*/*U*_eq_	
S1	0.28033 (7)	0.51733 (5)	0.34242 (3)	0.05338 (11)	
S2	0.69953 (8)	0.63001 (5)	0.25025 (3)	0.05403 (12)	
S3	0.61181 (7)	0.42429 (4)	0.11132 (3)	0.05159 (11)	
S4	0.85868 (7)	0.66560 (5)	0.02663 (3)	0.05264 (11)	
S5	1.22828 (7)	0.78128 (5)	0.09888 (4)	0.05328 (11)	
N1	0.6295 (2)	0.39444 (15)	0.39055 (10)	0.0471 (3)	
N2	0.8599 (2)	0.90383 (14)	0.11309 (11)	0.0448 (3)	
C1	0.2690 (2)	0.36628 (18)	0.43444 (11)	0.0437 (3)	
C2	0.4716 (2)	0.31514 (17)	0.45002 (11)	0.0433 (3)	
C3	0.5015 (3)	0.1931 (2)	0.52121 (15)	0.0561 (4)	
H3	0.634842	0.158220	0.532571	0.067*	
C4	0.3300 (3)	0.1255 (2)	0.57426 (15)	0.0611 (4)	
H4	0.348063	0.043560	0.621611	0.073*	
C5	0.1300 (3)	0.1771 (2)	0.55856 (14)	0.0617 (5)	
H5	0.016856	0.129419	0.596057	0.074*	
C6	0.0959 (3)	0.2970 (2)	0.48879 (14)	0.0560 (4)	
H6	−0.038178	0.330852	0.478180	0.067*	
C7	0.5497 (2)	0.49964 (18)	0.33205 (12)	0.0453 (3)	
C8	0.7176 (2)	0.56406 (15)	0.13211 (11)	0.0400 (3)	
C9	0.9663 (2)	0.79599 (16)	0.08213 (12)	0.0441 (3)	
C10	1.1930 (2)	0.93342 (17)	0.15548 (12)	0.0452 (3)	
C11	0.9856 (2)	0.98336 (15)	0.15608 (11)	0.0400 (3)	
C12	0.9199 (3)	1.10581 (17)	0.19921 (13)	0.0492 (3)	
H12	0.782958	1.140853	0.199684	0.059*	
C13	1.0611 (3)	1.17301 (19)	0.24069 (15)	0.0579 (4)	
H13	1.018665	1.253812	0.270076	0.070*	
C14	1.2667 (3)	1.1223 (2)	0.23954 (17)	0.0642 (5)	
H14	1.359240	1.169973	0.268185	0.077*	
C15	1.3362 (3)	1.0030 (2)	0.19685 (17)	0.0623 (5)	
H15	1.474206	0.969922	0.195698	0.075*	

### (sa) Atomic displacement parameters (Å^2^)

**Table:**

	*U*^11^	*U*^22^	*U*^33^	*U*^12^	*U*^13^	*U*^23^
S1	0.0471 (2)	0.0600 (3)	0.0492 (2)	0.00577 (17)	−0.00451 (16)	−0.00160 (17)
S2	0.0664 (3)	0.0563 (2)	0.04205 (19)	−0.02084 (19)	−0.00072 (17)	−0.01342 (17)
S3	0.0573 (2)	0.0457 (2)	0.0549 (2)	−0.00929 (17)	−0.00458 (17)	−0.01626 (17)
S4	0.0636 (3)	0.0529 (2)	0.04214 (19)	−0.01454 (18)	0.00143 (17)	−0.01137 (16)
S5	0.0464 (2)	0.0507 (2)	0.0647 (3)	0.00660 (16)	−0.00631 (18)	−0.01861 (19)
N1	0.0411 (6)	0.0555 (8)	0.0454 (7)	−0.0049 (5)	−0.0061 (5)	−0.0085 (6)
N2	0.0414 (6)	0.0412 (6)	0.0500 (7)	−0.0037 (5)	−0.0032 (5)	−0.0031 (5)
C1	0.0414 (7)	0.0533 (8)	0.0368 (6)	−0.0001 (6)	−0.0034 (5)	−0.0104 (6)
C2	0.0403 (7)	0.0512 (8)	0.0398 (7)	−0.0029 (6)	−0.0050 (5)	−0.0107 (6)
C3	0.0498 (9)	0.0597 (10)	0.0568 (9)	0.0001 (7)	−0.0107 (7)	−0.0010 (8)
C4	0.0674 (11)	0.0609 (11)	0.0515 (9)	−0.0092 (9)	−0.0069 (8)	0.0035 (8)
C5	0.0565 (10)	0.0774 (13)	0.0486 (9)	−0.0169 (9)	0.0034 (7)	−0.0048 (8)
C6	0.0412 (8)	0.0773 (12)	0.0479 (8)	−0.0053 (7)	−0.0005 (6)	−0.0082 (8)
C7	0.0459 (7)	0.0518 (8)	0.0396 (7)	−0.0065 (6)	−0.0033 (6)	−0.0116 (6)
C8	0.0399 (6)	0.0392 (7)	0.0414 (7)	0.0004 (5)	−0.0050 (5)	−0.0086 (5)
C9	0.0461 (7)	0.0412 (7)	0.0433 (7)	−0.0057 (6)	−0.0019 (6)	−0.0031 (6)
C10	0.0419 (7)	0.0450 (8)	0.0484 (8)	−0.0005 (6)	−0.0028 (6)	−0.0089 (6)
C11	0.0408 (7)	0.0359 (6)	0.0400 (6)	−0.0027 (5)	0.0003 (5)	−0.0003 (5)
C12	0.0485 (8)	0.0396 (7)	0.0558 (9)	0.0005 (6)	0.0021 (7)	−0.0052 (6)
C13	0.0664 (11)	0.0457 (8)	0.0616 (10)	−0.0068 (8)	0.0019 (8)	−0.0141 (7)
C14	0.0614 (11)	0.0643 (11)	0.0728 (12)	−0.0137 (9)	−0.0090 (9)	−0.0236 (9)
C15	0.0449 (9)	0.0680 (12)	0.0792 (13)	−0.0016 (8)	−0.0109 (8)	−0.0242 (10)

### (sa) Geometric parameters (Å, º)

**Table:**

S1—C1	1.7264 (17)	C3—C4	1.374 (3)
S1—C7	1.7368 (16)	C4—H4	0.9300
S2—C7	1.7610 (16)	C4—C5	1.389 (3)
S2—C8	1.7620 (15)	C5—H5	0.9300
S3—C8	1.6194 (15)	C5—C6	1.374 (3)
S4—C8	1.7468 (15)	C6—H6	0.9300
S4—C9	1.7685 (16)	C10—C11	1.400 (2)
S5—C9	1.7395 (16)	C10—C15	1.393 (2)
S5—C10	1.7299 (16)	C11—C12	1.402 (2)
N1—C2	1.391 (2)	C12—H12	0.9300
N1—C7	1.289 (2)	C12—C13	1.371 (3)
N2—C9	1.295 (2)	C13—H13	0.9300
N2—C11	1.383 (2)	C13—C14	1.390 (3)
C1—C2	1.404 (2)	C14—H14	0.9300
C1—C6	1.394 (2)	C14—C15	1.378 (3)
C2—C3	1.393 (2)	C15—H15	0.9300
C3—H3	0.9300		
			
C1—S1—C7	88.65 (8)	N1—C7—S2	123.20 (12)
C7—S2—C8	100.30 (7)	S3—C8—S2	126.96 (9)
C8—S4—C9	103.88 (7)	S3—C8—S4	117.64 (9)
C10—S5—C9	88.70 (7)	S4—C8—S2	115.36 (8)
C7—N1—C2	109.59 (13)	S5—C9—S4	119.69 (9)
C9—N2—C11	109.89 (13)	N2—C9—S4	123.63 (12)
C2—C1—S1	109.38 (12)	N2—C9—S5	116.68 (12)
C6—C1—S1	129.40 (13)	C11—C10—S5	109.30 (11)
C6—C1—C2	121.22 (16)	C15—C10—S5	129.31 (13)
N1—C2—C1	115.14 (14)	C15—C10—C11	121.39 (15)
N1—C2—C3	125.11 (15)	N2—C11—C10	115.42 (13)
C3—C2—C1	119.75 (15)	N2—C11—C12	125.09 (14)
C2—C3—H3	120.7	C10—C11—C12	119.49 (15)
C4—C3—C2	118.58 (17)	C11—C12—H12	120.6
C4—C3—H3	120.7	C13—C12—C11	118.85 (16)
C3—C4—H4	119.4	C13—C12—H12	120.6
C3—C4—C5	121.28 (18)	C12—C13—H13	119.5
C5—C4—H4	119.4	C12—C13—C14	121.06 (17)
C4—C5—H5	119.3	C14—C13—H13	119.5
C6—C5—C4	121.36 (17)	C13—C14—H14	119.3
C6—C5—H5	119.3	C15—C14—C13	121.41 (18)
C1—C6—H6	121.1	C15—C14—H14	119.3
C5—C6—C1	117.80 (16)	C10—C15—H15	121.1
C5—C6—H6	121.1	C14—C15—C10	117.79 (17)
S1—C7—S2	119.43 (10)	C14—C15—H15	121.1
N1—C7—S1	117.24 (12)		
			
S1—C1—C2—N1	0.14 (17)	C7—N1—C2—C1	−0.64 (19)
S1—C1—C2—C3	−179.90 (13)	C7—N1—C2—C3	179.42 (16)
S1—C1—C6—C5	179.91 (14)	C8—S2—C7—S1	−87.25 (10)
S5—C10—C11—N2	−0.09 (16)	C8—S2—C7—N1	97.19 (14)
S5—C10—C11—C12	179.50 (12)	C8—S4—C9—S5	104.26 (10)
S5—C10—C15—C14	−178.84 (16)	C8—S4—C9—N2	−74.91 (15)
N1—C2—C3—C4	−179.78 (17)	C9—S4—C8—S2	7.06 (11)
N2—C11—C12—C13	178.98 (15)	C9—S4—C8—S3	−174.93 (9)
C1—S1—C7—S2	−176.51 (10)	C9—S5—C10—C11	−0.52 (12)
C1—S1—C7—N1	−0.69 (13)	C9—S5—C10—C15	178.99 (19)
C1—C2—C3—C4	0.3 (3)	C9—N2—C11—C10	0.89 (18)
C2—N1—C7—S1	0.87 (17)	C9—N2—C11—C12	−178.67 (15)
C2—N1—C7—S2	176.52 (11)	C10—S5—C9—S4	−178.11 (10)
C2—C1—C6—C5	0.2 (3)	C10—S5—C9—N2	1.12 (13)
C2—C3—C4—C5	−0.5 (3)	C10—C11—C12—C13	−0.6 (2)
C3—C4—C5—C6	0.5 (3)	C11—N2—C9—S4	177.86 (11)
C4—C5—C6—C1	−0.4 (3)	C11—N2—C9—S5	−1.34 (17)
C6—C1—C2—N1	179.88 (15)	C11—C10—C15—C14	0.6 (3)
C6—C1—C2—C3	−0.2 (2)	C11—C12—C13—C14	0.6 (3)
C7—S1—C1—C2	0.26 (12)	C12—C13—C14—C15	0.0 (3)
C7—S1—C1—C6	−179.44 (17)	C13—C14—C15—C10	−0.6 (3)
C7—S2—C8—S3	2.17 (13)	C15—C10—C11—N2	−179.64 (16)
C7—S2—C8—S4	179.97 (9)	C15—C10—C11—C12	0.0 (2)

### (sa) Hydrogen-bond geometry (Å, º)

**Table:**

*D*—H···*A*	*D*—H	H···*A*	*D*···*A*	*D*—H···*A*
C6—H6···N1^i^	0.93	2.58	3.467 (2)	161
C15—H15···N2^ii^	0.93	2.68	3.552 (2)	157

Symmetry codes: (i) *x*−1, *y*, *z*; (ii) *x*+1, *y*, *z*.
